# MEK inhibition remodels the active chromatin landscape and induces SOX10 genomic recruitment in BRAF(V600E) mutant melanoma cells

**DOI:** 10.1186/s13072-019-0297-2

**Published:** 2019-08-09

**Authors:** Temesgen D. Fufa, Laura L. Baxter, Julia C. Wedel, Derek E. Gildea, NISC Comparative Sequencing Program, Stacie K. Loftus, William J. Pavan

**Affiliations:** 1 Genetic Disease Research Branch, National Human Genome Research Institute, National Institutes of Health, Bethesda, MD 20892 USA; 2 Ophthalmic Genetics and Visual Function Branch, National Eye Institute, National Institutes of Health, Bethesda, MD 20892 USA; 3 Computational and Statistical Genomics Branch, National Human Genome Research Institute, National Institutes of Health, Bethesda, MD 20892 USA; 4 NIH Intramural Sequencing Center, National Human Genome Research Institute, National Institutes of Health, Bethesda, MD 20892 USA

**Keywords:** Pigmentation, Melanocyte, MAPK pathway, MEK inhibitor, Enhancers, SOX10

## Abstract

**Background:**

The MAPK/ERK signaling pathway is an essential regulator of numerous cell processes that are crucial for normal development as well as cancer progression. While much is known regarding MAPK/ERK signal conveyance from the cell membrane to the nucleus, the transcriptional and epigenetic mechanisms that govern gene expression downstream of MAPK signaling are not fully elucidated.

**Results:**

This study employed an integrated epigenome analysis approach to interrogate the effects of MAPK/ERK pathway inhibition on the global transcriptome, the active chromatin landscape, and protein–DNA interactions in 501mel melanoma cells. Treatment of these cells with the small-molecule MEK inhibitor AZD6244 induces hyperpigmentation, widespread gene expression changes including alteration of genes linked to pigmentation, and extensive epigenomic reprogramming of transcriptionally distinct regulatory regions associated with the active chromatin mark H3K27ac. Regulatory regions with differentially acetylated H3K27ac regions following AZD6244 treatment are enriched in transcription factor binding motifs of ETV/ETS and ATF family members as well as the lineage-determining factors MITF and SOX10. H3K27ac-dense enhancer clusters known as super-enhancers show similar transcription factor motif enrichment, and furthermore, these super-enhancers are associated with genes encoding MITF, SOX10, and ETV/ETS proteins. Along with genome-wide resetting of the active enhancer landscape, MEK inhibition also results in widespread SOX10 recruitment throughout the genome, including increased SOX10 binding density at H3K27ac-marked enhancers. Importantly, these MEK inhibitor-responsive enhancers marked by H3K27ac and occupied by SOX10 are located near melanocyte lineage-specific and pigmentation genes and overlap numerous human SNPs associated with pigmentation and melanoma phenotypes, highlighting the variants located within these regions for prioritization in future studies.

**Conclusions:**

These results reveal the epigenetic reprogramming underlying the re-activation of melanocyte pigmentation and developmental transcriptional programs in 501mel cells in response to MEK inhibition and suggest extensive involvement of a MEK-SOX10 axis in the regulation of these processes. The dynamic chromatin changes identified here provide a rich genomic resource for further analyses of the molecular mechanisms governing the MAPK pathway in pigmentation- and melanocyte-associated diseases.

**Electronic supplementary material:**

The online version of this article (10.1186/s13072-019-0297-2) contains supplementary material, which is available to authorized users.

## Background

The mitogen-activated protein kinase (MAPK)/extracellular signal-regulated kinase (ERK) pathway is an evolutionarily conserved signaling cascade that relays multiple signals from the plasma membrane to the nucleus to regulate cell growth, differentiation, proliferation, and survival. In MAPK/ERK pathways, ligand binding to a receptor tyrosine kinase at the plasma membrane leads to activation of the small GTPase RAS. This is followed by a cascade of complex protein interactions and phosphorylation events, including RAF phosphorylation and activation of MAPK kinase (MEK), which directly phosphorylates ERK and regulates its nuclear translocation and transcriptional activity [1, 2]. MAPK pathway deregulation due to genomic mutations is common in many human cancers, including thyroid cancer, colon cancer, serous ovarian cancer, and melanoma [3, 4]. Approximately 50% of melanomas carry the activating BRAF(V600E) mutation, which causes constitutive hyperactivation of the MAPK pathway [5]. Small-molecule inhibitors of BRAF such as vemurafenib and dabrafenib have shown remarkable results as molecularly targeted therapies for melanoma, especially when used in combination with MEK inhibitors, including the MEK inhibitor AZD6244 (also known as selumetinib or ARRY-142886) [6–14]. However, clinical responses to BRAF and MEK inhibitors are often not durable due to acquisition of resistance after treatment [15–17], highlighting the need to identify novel melanoma vulnerabilities that could be exploited for new treatments.

One approach to address this need is through analysis of epigenetic changes that arise from altered MAPK/ERK pathway signaling. Chromatin-mediated processes play crucial roles in the signaling cascades that govern development, cancer progression, and drug resistance [18–20]; however, little is known about the chromatin signatures and epigenetic effects caused by MAPK inhibitors. In particular, altered chromatin modifications at enhancers—non-coding DNA elements that interact with transcription factors and chromatin remodelers—have been implicated in aberrant modulation of gene expression and drug resistance in many cancers, including melanoma [21–26]. Enhancers can be identified through profiling of specific biochemical signatures of chromatin, such as accessibility to transposase digestion, hypersensitivity to DNase I cleavage, and acetylation of histone H3 on lysine residue 27 (H3K27ac, [27–29]). In addition, large clusters of enhancers called super-enhancers have been implicated in the regulation of normal development [30, 31] as well as cancer progression and resistance to therapy [24, 32, 33]. Super-enhancers can undergo remodeling during development, in response to disease, or as a result of functional genomic and pharmacological perturbations, and correspondingly super-enhancers regulate genes that govern cell identity, development, and pathogenesis [24, 25, 30–32, 34–36]. Super-enhancers exhibit an exceptionally high density of binding for numerous factors, including the multiprotein Mediator complex and BRG chromatin remodeling complex, transcriptional cofactors, and lineage-determining transcription factors, including members of the SOX transcription factor family in embryonic stem cells (SOX2), neural progenitor cells (SOX2), chondrocytes (SOX9, SOX5/6), and hair follicle stem cells (SOX9) [30, 31, 37–39].

The SOX family member SOX10 plays a crucial role in neural crest and melanocyte development as well as human disease. SOX10 mutations are associated with the human neurocristopathies Waardenburg Syndrome (types 2 and 4) and PCWH (OMIM#609136, #611584, and #613266, https://omim.org/), in which abnormal neural crest/melanocyte development results in phenotypes that include hypopigmentation and enteric ganglia defects. SOX10 predominantly occupies distal (non-promoter) enhancer regions in melanocyte and melanoma genomes, colocalizes with MITF at enhancers, and interacts with BRG1 at melanocyte-specific enhancer regions [18, 40–44]. Furthermore, SOX10 is required for melanoma initiation, survival and progression, and cellular levels of SOX10 affect acquired resistance to melanoma inhibitor drugs [45–49].

In this study, we used a BRAF(V600E) mutant melanoma cell line as a model system to evaluate genome-wide changes in gene expression, enhancer chromatin marks, and transcription factor occupancy in response to the MEK inhibitor AZD6244. AZD6244 treatment caused hyperpigmentation as well as widespread alterations in transcription and the active chromatin landscape that were associated with numerous pigmentation genes. Distinct transcriptional motifs underlying AZD6244-induced epigenome remodeling were discovered, including SOX, MITF, ETV, and ATF motifs. A novel cohort of super-enhancers was identified that was enriched in melanocyte lineage-specific transcription factor motifs. Extensive recruitment of SOX10 occurred throughout the genome under MEK inhibition, including increased interactions at H3K27ac-associated enhancers and super-enhancers, with notable binding of SOX10 at super-enhancers associated with AZD6244-upregulated pigmentation genes. Furthermore, many of these chromatin regions directly overlapped GWAS loci for human pigmentation phenotypes. These data provide a roadmap of enhancer activity in a BRAF(V600E) mutant cell line under MEK inhibition, suggest the chromatin regions identified in this study regulate pigment- and developmentally related programs of gene expression, and implicate SOX10 in the regulation of these responses to MEK inhibition.

## Results

### MEK inhibition induces pigmentation in melanoma cells

**Fig. 1:**
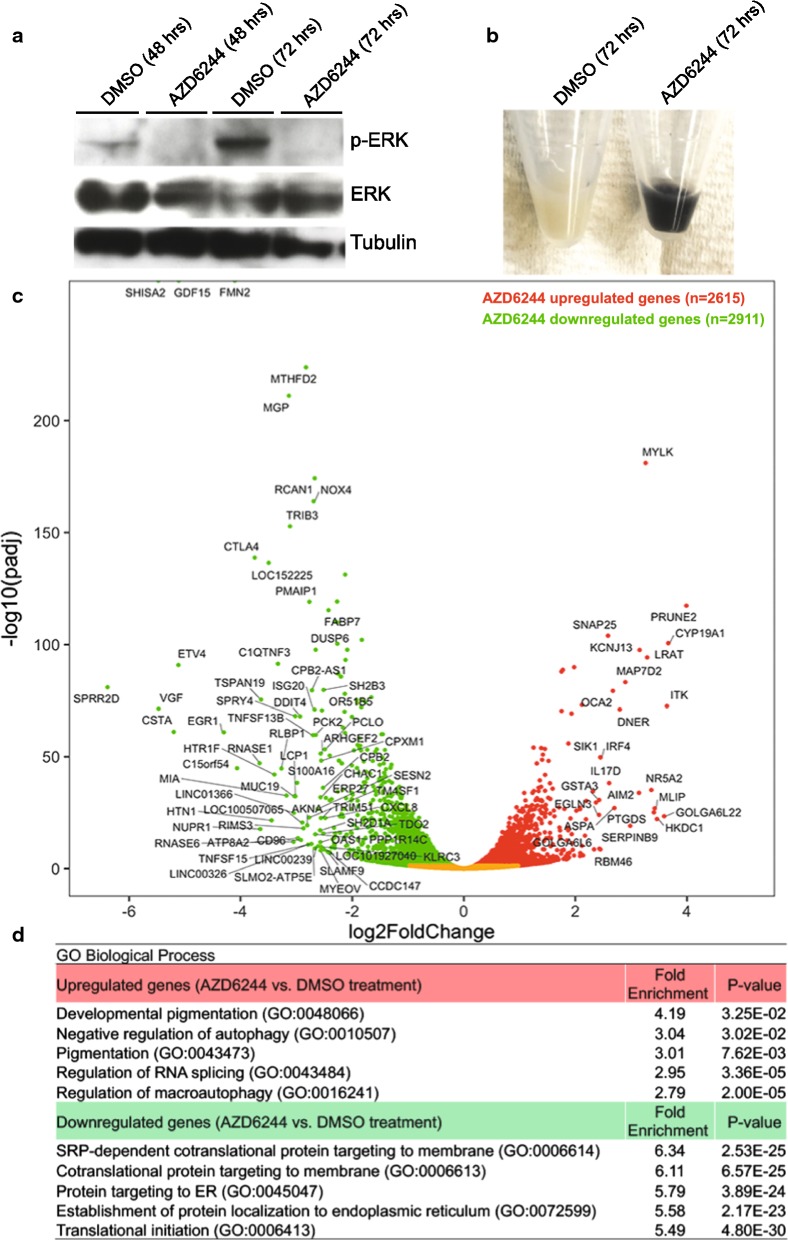
Inhibition of MEK induces hyperpigmentation and widespread gene expression changes in 501mel melanoma cells. **a** ERK and phosphorylated ERK (p-ERK) Western blots of whole-cell lysates derived from 501mel cells treated with DMSO or AZD6244 (200 nM). Tubulin was used as a protein loading control. **b** Cell pellets from 501mel cells after 72 h of treatment with DMSO or AZD6244. **c** Volcano plot of genes with significantly altered expression levels under AZD6244 treatment. Downregulated genes are in green, and upregulated genes are in red; the genes with an absolute fold-change > 2.4 and a *p*-adjusted value > 0.05 are labeled. **d** Gene ontology analysis on the genes significantly upregulated and downregulated in response to AZD6244 treatment. The top enriched processes for upregulated genes affect developmental pigmentation and autophagy, while those for downregulated genes affect protein targeting to membranes and protein translation

To assess the impact of MEK inhibition on the epigenome in melanoma cells, MEK inhibitor-responsive human 501mel cells (heterozygous for the BRAF(V600E) mutation) were treated with AZD6244 (MEK inhibitor) or DMSO (vehicle control). As expected, treatment of 501mel cells with AZD6244 for 48 h or 72 h eliminated ERK phosphorylation (Fig. 1a); however, the cells remained proliferative (data not shown). Interestingly, 501mel cells became hyperpigmented following AZD6244 treatment, as evidenced by the dramatic change in color of the cell pellets (Fig. 1b). Similar hyperpigmentation effects of AZD6244 treatment were observed in two additional melanoma cell lines (A375 and mouse B16F10 melanoma cell lines, Additional file 1: Figure S1). The emergence of AZD6244-induced hyperpigmentation in melanoma cells suggested that melanocyte lineage-specific pathways may be re-activated in response to MEK inhibition.

Gene expression profiling by RNA-Seq was performed to identify genes whose expression changed in response to AZD6244 treatment in 501mel cells. Significant expression changes (false discovery rate (FDR)-adjusted *p* value < 0.05) were identified for 5526 genes following AZD6244 treatment compared to control cells (Fig. 1c), with similar numbers of upregulated (*n* = 2615) and downregulated (*n* = 2911) genes (Additional file 2: Table S1). Gene ontology (GO) analysis (geneontology.org, [50]) found distinct differences between the upregulated and downregulated genes (Fig. 1d). The upregulated genes showed enrichment of developmental pigmentation and autophagy processes, consistent with the reappearance of AZD6244-induced pigmentation (Fig. 1b). Correspondingly, 250 of the 5526 differentially expressed genes were known to affect pigmentation phenotypes in human, mouse, or zebrafish ([51], Additional file 2: Table S2), suggesting widespread alteration of pigmentation pathways in response to AZD6244 treatment. These genes included two of the genes with the largest increase in expression, *OCA2* and *IRF4* (Fig. 1c), which affect albinism and normal human pigmentation variation [52–55]. In contrast, downregulated genes were enriched for processes affecting protein targeting to membranes and protein translation, suggesting widespread attenuation of these pathways. These processes were driven by a cohort of 81 genes that encompassed 40S and 60S ribosomal proteins, signal recognition particle pathway proteins, and ribosomal cofactors, consistent with MAPK pathways regulating the ribosomal biogenesis necessary for proliferation and survival [56].

### MEK inhibition remodels the active chromatin landscape

**Fig. 2:**
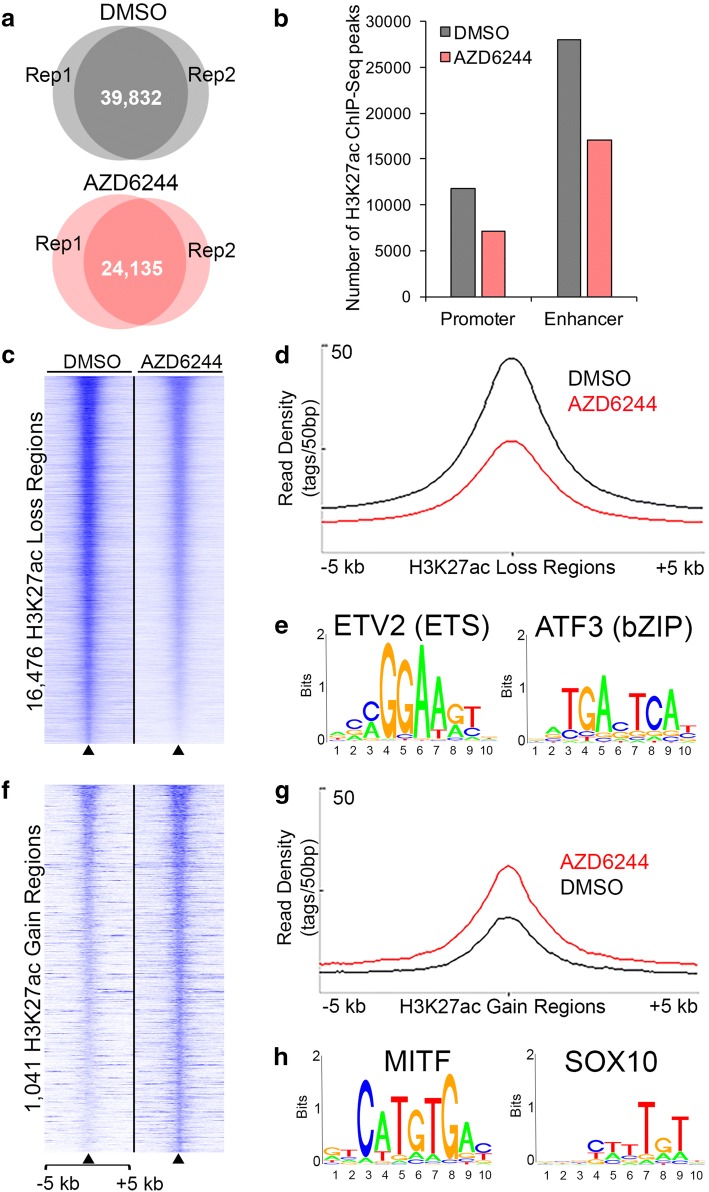
MEK inhibition induces genome-wide modulation of the active chromatin landscape in 501mel cells. **a** Venn diagrams showing overlap of H3K27ac peaks identified by ChIP-Seq in independent replicate samples of 501mel cells treated with DMSO (upper panel) and AZD6244 (lower panel). **b** Distribution of H3K27ac peaks in 501mel cells treated with DMSO (gray) or AZD6244 (red) at either promoter (< 2.5 kb from TSS) or non-promoter, distal enhancer regions. **c**, **f** Heatmaps showing relative H3K27ac signal in 501mel cells treated with DMSO or AZD6244. H3K27ac ChIP-Seq tag densities are shown for significantly differentially remodeled H3K27ac sites (FDR < 0.05) at regions **c** losing H3K27ac or **f** gaining H3K27ac. Each row represents a 10-kb region centered on differentially remodeled H3K27ac regions, sorted by H3K27ac signal enrichment. **d**, **g** Line plots show average H3K27ac ChIP-Seq signal intensities (*y*-axis) across regions **d** losing H3K27ac or **g** gaining H3K27ac over a ± 5 kb genomic window centered on differentially remodeled H3K27ac sites (*x*-axis). **e**, **h** The top significantly enriched transcription factor motifs identified by de novo motif discovery of sequences in regions **e** losing H3K27ac and **h** gaining H3K27ac. The top 5 enriched motifs, the fraction of H3K27ac regions containing the motifs, and the *p* values for the overrepresentation of each motif are shown in Additional file 2: Table S3

To evaluate the broad chromatin changes underlying the cellular response to MEK inhibition, chromatin immunoprecipitation followed by high-throughput sequencing (ChIP-Seq) was performed for H3K27ac, an established histone modification mark of transcriptionally active regulatory elements such as promoters and enhancers [28, 29]. Treatment of 501mel cells with AZD6244 for 72 h resulted in a decrease in the total number of H3K27ac-enriched regions (peaks) relative to DMSO-treated (control) cells; 39,832 and 24,135 significantly enriched H3K27ac peaks were detected in control and AZD6244 groups, respectively (FDR < 0.05, Fig. 2a). In both control and AZD6244-treated samples, the majority of H3K27ac-enriched regions were located outside of proximal promoter regions (Fig. 2b), consistent with the known enrichment of H3K27ac in distal enhancer regions [28, 29]. The genome-wide reduction of H3K27ac in response to AZD6244 treatment was also reflected in the complete loss of 22,634 H3K27ac peaks, as compared to 1989 novel, newly gained H3K27ac regions.

#### MEK inhibitor-induced, differentially enriched H3K27ac regions contain distinct transcriptional motif signatures

DiffBind [57] was used to quantitatively compare H3K27ac peaks that were present in both DMSO-treated and AZD6244-treated cells, but exhibited differential enrichment of H3K27ac levels following AZD6244 treatment. This analysis found that 17,517 regions across the genome exhibited significant differential enrichment of H3K27ac (FDR < 0.05). More than 76% of these regions were located outside the proximal promoters of their putative target genes (> 5 kb from transcriptional start sites, TSS), again consistent with H3K27ac enrichment at distal regulatory elements/enhancers. Functional enrichment analysis of the top ~ 20% of differentially enriched H3K27ac regions (|FC| > 1.456, *n* = 3359) using the Genomic Region Enrichment of Annotation Tool (GREAT, [58]) showed enrichment of biological processes related to growth, differentiation, and development. The vast majority of the 17,517 differently enriched regions showed significantly decreased H3K27ac (16,476 regions, Fig. 2c, d), while the remaining 1041 regions exhibited significantly increased H3K27ac (Fig. 2f, g). Interestingly, identification of differentially expressed genes (Additional file 2: Table S1) that are also putative targets of the 17,517 H3K27ac-marked enhancers (identified using GREAT) showed similar numbers of both up-and downregulated genes in enhancer regions that were losing H3K27ac (1623 upregulated and 1993 downregulated genes) and in regions gaining H3K27ac (350 upregulated and 189 downregulated genes). This suggests that H3K27ac loss does not exclusively cause downregulation of gene expression, nor does H3K27ac gain only cause upregulation of gene expression.

H3K27ac-enriched chromatin regions harbor active enhancer elements and binding sites for transcription factors [28, 59–61]. To identify the transcriptional motif signatures underlying differentially enriched H3K27ac regions in AZD6244-induced MEK inhibition, the HOMER motif analysis algorithm [62] was applied to the DNA sequences at differentially enriched H3K27ac regions. This analysis revealed enrichment of motif signatures of signal/stress-dependent regulatory proteins in the regions losing H3K27ac, where the top two enriched motifs were ETV and ATF binding motifs (Fig. 2e, Additional file 2: Table S3). Interestingly, numerous ETV and ATF transcription factor family members with similar consensus motif recognition showed significantly decreased mRNA expression under AZD6244 treatment, including *ETV1*, *ETV4*, *ETV5*, *ATF3*, and *ATF4* (Fig. 1c, Additional file 2: Table S1), suggesting MEK inhibition may attenuate transcriptional programs regulated by these factors. In contrast, the highest ranked motifs in regions that gained H3K27ac corresponded to E-box and SOX consensus motifs (Fig. 2h, Additional file 2: Table S3). E-box and SOX motifs are recognized by the lineage-determining factors MITF and SOX10, respectively, which are essential for melanocyte development and differentiation, normal melanocyte function, and melanoma initiation and proliferation [45, 63–67]. In addition, *SOX10*, *SOX4*, *SOX11*, and *MITF* all showed significantly increased mRNA expression under AZD6244 treatment, whereas *SOX5*, which acts to inhibit SOX10 activation of downstream targets in melanocyte development [68], was significantly downregulated (Additional file 2: Table S1). Therefore, the expression changes in these transcription factors are consistent with the re-activation of developmental and pigmentation pathways.

### Lineage-specific and pigmentation genes are associated with differentially acetylated H3K27ac regions

Given the enriched motifs for the melanocyte regulatory transcription factors MITF and SOX10, functional enrichment analyses using GREAT were performed to assess the corresponding genes associated with differentially enriched H3K27ac regions. The GREAT-identified genes for all 17,517 of the differentially enriched regions included 195 (78%) of the 250 pigmentation genes that show significant expression changes under MEK inhibition (Additional file 2: Table S2). The subset of genes associated with the 1041 regions gaining H3K27ac showed enrichment for pathways regulating morphogenesis and development, including oligodendrocyte differentiation, the phosphatidylinositol 3-kinase cascade, regulation of gliogenesis, and cell-fate specification (Additional file 1: Figure S2). These pathways are intriguing because melanocytes and glia of the peripheral nervous system (Schwann cells) are both derived from the neural crest lineage, and also because SOX10 is known to regulate lineage-specific/developmental pathways in melanocytes, glia, and oligodendrocytes [69–73]. In contrast, genes associated with the 16,476 regions losing H3K27ac showed significant enrichment of pathways regulating protein localization and protein targeting to membranes and endoplasmic reticulum (Additional file 1: Figure S2). Taken together, these data suggest the H3K27ac-marked regions may regulate lineage-specific, developmental, and pigmentation programs in response to MEK inhibition.

### Identification of super-enhancers in 501mel cells and their association with pigment genes

**Fig. 3:**
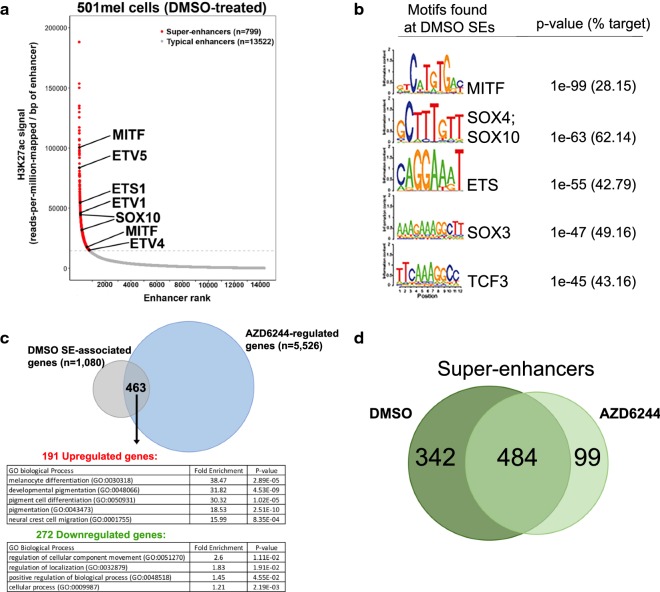
Identification of super-enhancers in 501mel cells and analysis of super-enhancer-associated gene response to MEK inhibition. **a** Distribution of H3K27ac ChIP-Seq signal at enhancers (input-subtracted reads per million per enhancer region) in 501mel cells under DMSO treatment. Enhancers are ranked by decreasing H3K27ac ChIP-Seq signal; the gray dashed line demarcates the boundary between super-enhancers (red) and typical enhancers (gray). The transcription factors listed exhibit 3 notable characteristics: each of their genomic loci are associated with nearby super-enhancers (indicated by black lines), each show differential mRNA expression under AZD6244 treatment (shown in Additional file 2: Table S1), and each also have enriched motifs when all super-enhancer regions are examined (shown in **b**). Therefore, these transcription factors are not only associated with a neighboring super-enhancer, but themselves have the capacity to bind other super-enhancers throughout the genome, raising the possibility of autoregulatory loops involving these key factors. Of note, MITF is shown twice because the *MITF* genomic locus has two independent super-enhancers. **b** The top five transcription factor binding motifs significantly enriched in 501mel super-enhancers; the fraction of super-enhancer regions containing the indicated motifs and the *p* values for the overrepresentation of each motif are shown. **c** Venn diagram showing the overlap between all genes associated with super-enhancers and genes whose expression is significantly altered by AZD6244 in 501mel cells. GO analysis was performed using the PANTHER overrepresentation test, to identify top biological pathways enriched for upregulated (upper panel, red) and downregulated (lower panel, green) genes with super-enhancer signatures in 501mel cells. *p* values represent FDR-adjusted (Bonferroni method) significance of enrichment for pathway members. **d** Venn diagram showing the overlap of super-enhancers in control (DMSO-treated) and AZD6244-treated 501mel cells

Super-enhancers are characterized by extremely high densities of H3K27ac and transcription factors and regulate genes involved in development and disease [24, 30–33]. To define the constitutive super-enhancers found in 501mel cells, the ROSE algorithm [30, 31] was applied to the H3K27ac ChIP sequences from control (DMSO-treated) 501mel cells; this approach identified 13,523 typical enhancers and 799 super-enhancers (Fig. 3a, Additional file 2: Table S4). DNA motif enrichment analysis was performed on the 799 super-enhancers to identify putative interacting transcription factors, and this revealed significant enrichment of binding motifs for MITF, SOX4/SOX10, and ETS (Fig. 3b), transcription factors that are essential for melanocyte lineage development, differentiation, and melanomagenesis [63, 65–67].

The ROSE program was used to link the 799 super-enhancers to their putative target genes, identifying 1080 super-enhancer-associated genes. GO analysis of these super-enhancer-associated genes revealed top enriched biological processes regulating melanocyte differentiation and pigmentation, as well as neural crest development, adhesion, and migration (Additional file 2: Table S5). Comparison of the 1080 super-enhancer-associated genes with the 5526 differentially expressed genes (Additional file 2: Table S1) identified a set of 463 genes that were both super-enhancer-associated and exhibited altered expression under MEK inhibition (Fig. 3c). Interestingly, these 463 genes included transcription factors whose binding motifs are enriched in the 799 super-enhancers, including *MITF*, *SOX10*, and *ETS1* (Fig. 3a). This suggests that these transcription factors have the potential to act at their associated super-enhancers in autoregulatory feedback loops. Furthermore, the upregulated subset of the 463 genes was enriched in biological pathways linked to neural crest/melanocyte development and pigmentation, in contrast to the broad regulatory pathways affecting cellular component movement and localization associated with the downregulated gene subset (GREAT analysis, Fig. 3c). Correspondingly, 45 of these 463 differentially expressed, super-enhancer-associated genes are known pigmentation genes, and 71% (32 of 45) of these pigment genes were upregulated under AZD6244 treatment (Additional file 2: Table S6). Taken together, these data support a role for these super-enhancers in regulating pigmentation and melanocyte/neural crest-related developmental pathways in 501mel cells.

### Super-enhancers in AZD6244-treated 501mel cells lose H3K27ac but retain similar genomic locations

Using the same approach employed for identifying super-enhancers in control 501mel cells, we identified 583 super-enhancers and 721 super-enhancer-associated genes in AZD6244-treated 501mel cells (Additional file 1: Figure S3, Additional file 2: Table S4). Along with a reduced total number of super-enhancers (583 vs. 799), H3K27ac signal density at super-enhancers was significantly decreased under AZD6244 treatment (Additional file 1: Figure S3), which was reflected in an overall decrease in enhancer scores and was consistent with MEK inhibitor-induced loss of H3K27ac throughout the genome (Fig. 1a). Interestingly, while many control super-enhancers were no longer present following AZD6244 treatment (42%; 342 out of 799), the majority of super-enhancers identified in AZD6244-treated cells overlapped with control super-enhancers (83%; 484 out of 583, Fig. 3d), indicating retention of most super-enhancers at similar genomic locations following MEK inhibition. Correspondingly, 85% of differentially expressed genes associated with AZD6244 super-enhancers were the same as the control 463 gene set (264 out of 311 genes), including persistence of the motif-enriched transcription factors *SOX10*, *MITF*, *ETV5,* and *ETS1* (Additional file 1: Figure S3). Notably, this overlap also included super-enhancers linked to pigmentation genes, as 28 of the 45 super-enhancers associated with differentially expressed pigmentation genes persisted under AZD6244 treatment (Additional file 2: Table S6).

### AZD6244 treatment stimulates widespread recruitment of SOX10 to chromatin

SOX10 plays critical roles in melanocyte development and function, including direct transcriptional regulation of a subset of pigmentation genes [74–81]. Having demonstrated that MEK inhibition induces pigmentation in 501mel cells, and that SOX binding motifs are overrepresented at enhancer and super-enhancer regions, we used ChIP-Seq analysis to examine the genomic binding of SOX10. DNA motif enrichment analysis of SOX10 ChIP-Seq peaks revealed significant enrichment of both dimer and monomer SOX consensus motifs in control and AZD6244-treated cells (Additional file 1: Figure S5). The regions directly flanking SOX10 ChIP-Seq peak summits (both in control and AZD6244-treated cells) also contained overrepresented sequences for binding motifs of HBP1 and LEF1, transcriptional regulators of the WNT/β-catenin pathway, as well as E-box binding motifs for the transcription factors TFE3, TFEB, and MITF (Additional file 1: Figure S5).

**Fig. 4:**
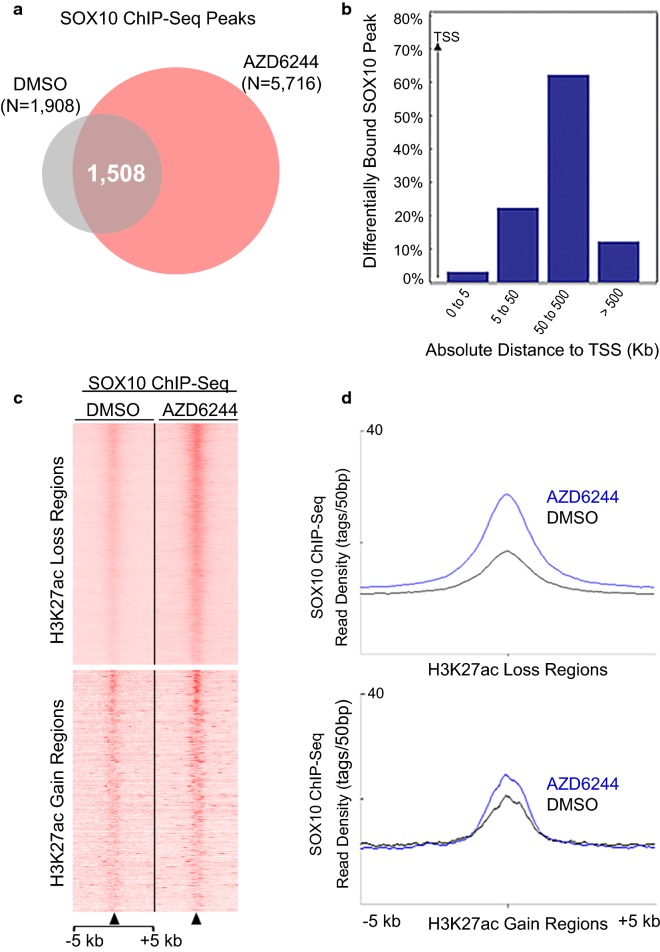
MEK inhibition induces extensive SOX10 chromatin binding in 501mel cells. **a** Venn diagram showing overlap of SOX10 ChIP-Seq peaks in DMSO- and AZD6244-treated 501mel cells. In both treatment groups, the number of SOX10 ChIP-Seq peaks represents the total that were replicated across two independent experiments. Significance cutoff for SOX10 ChIP-Seq peaks was *p* < 1e10^−5^. **b** Genomic distribution of differentially bound SOX10 peaks in 501mel cells (AZD6244 vs DMSO) with respect to the nearest TSS as revealed by GREAT (using the basal plus extension settings). Values on the *Y*-axis represent percent of peaks found in a given genomic bin relative to TSS, i.e., [0, 5 kb], [5 kb, 50 kb], [50 kb, 500 kb], [500 kb, infinity]. **c** Heatmap of SOX10 ChIP-Seq signals at genomic regions that lost H3K27ac (upper panel) or gained H3K27ac (lower panel) in response to AZD6244 treatment (in units of read per million of genomic region). Each row shows a ± 5 kb region centered on an H3K27ac region, and rows are ordered by SOX10 ChIP-Seq signal. **d** Line plots showing average SOX10 ChIP-Seq signal intensities (*y*-axis) across regions losing H3K27ac (upper panel) and gaining H3K27ac (lower panel) over a ± 5 kb genomic window centered on differentially remodeled H3K27ac sites (*x*-axis)

Remarkably, SOX10 showed a massive increase in binding sites in response to AZD6244 treatment: 5716 discrete peaks were occupied by SOX10 in AZD6244-treated cells, which is a nearly threefold increase in comparison with the 1908 SOX10 peaks identified in control cells (Fig. 4a). Most SOX10 peaks in control cells were retained in AZD6244-treated samples (1508, 79% of control peaks). Quantitative comparisons of SOX10 ChIP-Seq tag density in control and AZD6244-treated samples using DiffBind showed that 4602 peaks were differentially occupied by SOX10; over 70% of these are located more than 50 kb away from the TSSs of neighboring genes (Fig. 4b), as expected from the previously described distal enhancer binding profile of SOX10 [40].

Given the extensive genome-wide recruitment of SOX10, we examined the H3K27ac-marked enhancers that exhibited novel SOX10 binding in response to AZD6244 treatment, and found enriched SOX10 binding at H3K27ac-marked regions. A total of 1935 enhancers showed novel SOX10 binding sites in AZD6244-treated cells, a more than threefold increase over the 526 SOX10-bound enhancers that were only seen in control (DMSO-treated) cells. In addition, examination of SOX10 binding at the 17,517 differentially enriched H3K27ac regions showed increased SOX10 binding under AZD6244 treatment, with greater SOX10 binding at regions with reduced H3K27ac (Fig. 4c, d, Additional file 1: Figure S4). SOX10 binding at super-enhancers was also increased under MEK inhibition. In control cells, 36% of super-enhancers (285 out of 799) contained SOX10 ChIP binding sites, while in AZD6244-treated cells the super-enhancers containing SOX10 binding increased to 66% (385 of 583). A detailed description of the dynamic SOX10 binding seen at all H3K27ac-marked regions in control and AZD6244-treated cells is contained in Additional file 1: Figure S4. The enrichment of SOX10 binding at the H3K27ac-marked enhancers and super-enhancers under AZD6244 treatment suggests that SOX10 may function to regulate the pathways associated with these regions by GO analysis, which included melanocyte differentiation and pigmentation, neural crest development, adhesion, and migration.

### SOX10 binds at enhancers near pigmentation genes

GREAT analysis on the 1935 enhancers with novel SOX10 binding showed top enriched biological processes of cell chemotaxis and pigmentation; correspondingly, many of the H3K27ac-marked regions with enriched SOX10 binding were associated with pigmentation genes that showed significant expression changes under AZD6244 treatment. Out of the 250 differentially expressed pigmentation genes associated with H3K27ac-enriched regions, 76 genes showed SOX10 binding at their associated H3K27ac-enriched regions under AZD6244 treatment (Additional file 2: Table S2), with 62 of these genes showing novel SOX10 binding sites. (The remaining 14 SOX10 binding sites were constant between DMSO- and AZD6244-treatment.) In contrast, only 21 genes showed novel SOX10 binding to their associated H3K27ac-enriched regions under control conditions. The H3K27ac-enriched regions with SOX10 binding included super-enhancers: 21 of the 29 super-enhancers associated with differentially expressed pigmentation genes under AZD6244 treatment were bound by SOX10 (72%), 18 of which exhibited novel SOX10 recruitment under AZD6244 (Additional file 2: Table S6). Furthermore, the majority of the pigmentation genes associated with these SOX10-bound super-enhancers showed upregulated gene expression under AZD6244 treatment (86%, Additional file 2: Table S6). This upregulated gene set included *MITF*, *SNAI2*, *ZEB2, ETS1,* and *TFAP2A*, all critical transcription factors of the melanocyte lineage (Additional file 1: Figure S6).

To assess SOX10’s functional role in AZD6244-induced pigmentation, we reduced SOX10 protein levels in 501mel cells by the stable transfection of two independent shSOX10 constructs, each in triplicate. Complete ablation of SOX10 in melanoma cells induces growth arrest and senescence [46], and consistent with this finding SOX10 protein was partially reduced by all 6 shSOX10-expressing cell lines (Additional file 1: Figure S7). When treated with AZD6244, all shSOX10-expressing cell lines became pigmented, similar to control cells stably transfected with a non-targeting shRNA (Additional file 1: Figure S7), indicating that the partial reduction in SOX10 levels that occurred in these cells did not prevent the ability of AZD6244 to induce pigmentation.

### GWAS comparisons support functional relevance of active chromatin regions to pigmentation-associated genetic variants

The genetic variants for human diseases and traits identified from genome-wide association studies (GWAS) primarily occur in non-coding genomic regions [19, 82, 83]. We therefore wanted to determine which GWAS single nucleotide polymorphisms (SNPs) associated with melanocyte phenotypes resided within our collection of melanocyte lineage distal enhancer elements. The H3K27ac-marked regions in 501mel cells were compared to 814 SNPs that have been previously implicated in human pigmentation and/or melanoma through GWAS [84–86]. These comparisons identified 90 pigmentation/melanoma GWAS loci that overlap with regions of H3K27ac acetylation and/or SOX10 ChIP-Seq binding (Table 1, Additional file 2: Table S7), thus highlighting the variants located within these regions as high priority candidates for future studies of the function of these distal enhancers. The associated phenotypes included normal pigmentation of hair, skin, and eye as well as suntan or sunburn, nevus formation, and cutaneous melanoma (Table 1).

**Table 1:** Overlap of 90 pigmentation and melanoma GWAS SNPs with H3K27ac-marked regions and with SOX10 ChIP binding

Genomic regions	Overlapping GWAS SNPs	GWAS-associated phenotypes
H3K27ac-marked regions (DMSO or AZD6244)	70	Hair color, eye color, skin pigmentation, cutaneous melanoma, nevus, freckles, suntan, sunburn, vitiligo
H3K27ac differentially acetylated regions	52	Hair color, eye color, skin pigmentation, cutaneous melanoma, nevus, freckles, suntan, vitiligo
H3K27ac super-enhancers (DMSO or AZD6244)	46	Hair color, eye color, skin pigmentation, cutaneous melanoma, nevus, freckles, suntan, vitiligo
SOX10 ChIP (DMSO or AZD6244)	8	Hair color, eye color, skin pigmentation, cutaneous melanoma, freckles, suntan, sunburn, vitiligo

## Discussion

MAPK/ERK signaling is crucial for regulation of proliferation, differentiation, and pigmentation of melanocytes, and abnormal MAPK/ERK signaling leads to uncontrolled cell proliferation and melanoma tumorigenesis [87, 88]. We used the BRAF(V600E) mutant 501mel human cell line as a model system to discover the chromatin alterations that underlie the re-activation of melanocyte-intrinsic pigmentation in the context of MEK inhibition. Our chromatin-based approach showed that MEK inhibition re-activates melanocyte lineage-specific transcriptional programs, consistent with a previous study suggesting re-activation of lineage-specific programs under MEK inhibition [89]. Our results also showed that MEK inhibition extensively alters the active chromatin landscape and induces widespread SOX10 binding to additional genomic targets.

Our analysis used H3K27ac, a marker of active regulatory DNA elements including enhancers, to reveal the epigenetic and transcriptional alterations underlying MEK inhibition. AZD6244 treatment resulted in a dramatic reduction in genomic H3K27ac levels, with over 20,000 regions completely lost, and reduced H3K27ac levels at 94% of the 17,517 enhancer regions with significant acetylation changes. We also examined super-enhancers, because of their predicted role in governing the expression of genes affecting cell development/identity as well as oncogenic pathways [30–32]. We identified 799 super-enhancers in 501mel cells, showed that a subset of these remain constant under MEK inhibition, and also found that over 50% of these are remodeled in a MAPK-dependent manner, again with widespread loss in H3K27ac acetylation levels. Intriguingly, the loss of H3K27ac did not result in widespread loss of gene expression, as might be expected since H3K27ac marks transcriptionally active chromatin [28, 29], but instead resulted in relatively equal numbers of genes with increased and decreased expression. This suggests 501mel cells show a complex transcriptional response to the extensive H3K27ac deacetylation induced by MEK inhibition that is not simply a widespread attenuation of gene expression. The enhancers and super-enhancers identified here and their associated changes under MEK inhibition provide a window into the tremendous genome-wide reorganization that occurs at these regulatory genome features.

Enriched motif analysis of these enhancer and super-enhancer regions suggested that transcription factors with the capacity to bind ETV/ETS, ATF, SOX, E-BOX, and TCF motifs may play a role in regulation of these regions. Interestingly, several factors that bind to these overrepresented motifs are expressed in 501mel cells and either upregulated (*SOX10*, *SOX4*, *SOX11*, and *MITF*) or downregulated (*SOX5*, *ETV1*, *ETV4*, *ETV5*, *ATF3*, and *ATF4*) in response to MAPK inhibition. These results correlate with previous studies showing that MITF, ETS1, and ETV1 are downstream effectors of MAPK signaling in normal melanocytes and in melanoma progression [90–93]. Interestingly, the genomic loci for *SOX10, MITF, ETS1*, *ETV1*, *ETV4*, and *ETV5* are associated with super-enhancers, suggesting the possibility for autoregulation; future studies will be needed to determine the roles for each of these transcription factor candidates in regulating these enhancers and super-enhancers in melanocytes and melanoma. Importantly, our study also demonstrates that SOX10 directly binds to numerous H3K27ac-marked regions in 501mel cells under MEK inhibition, consistent with SOX10 acting as a downstream effector of the MAPK pathway.

SOX10 is a member of the SOX family of transcription factors, which all exhibit strong transcriptional regulation of developmental processes and cell-fate specification in a lineage-specific manner [94, 95]. Recent studies have provided evidence that SOX10 occupies a key regulatory node in the response to MAPK signaling in the nucleus: SOX10 emerged from a shRNA screen for genes that confer resistance to BRAF and MEK inhibitors via upregulation of EGFR and PDGFRB [47], and SOX10 is directly phosphorylated by ERK as part of a regulatory axis involving ERK1/2, SOX10, FOXD3, and ERBB3 that mediates adaptive resistance to RAF inhibitors in BRAF mutant melanoma cells [49]. Our analysis now provides additional data for SOX10 acting downstream of the MAPK signaling cascade: the epigenetic reconfiguration of the genome under MEK inhibition included both a dramatic increase in genome-wide SOX10 chromatin occupancy and extensive SOX10 binding at enhancer and super-enhancer regions with decreased H3K27ac levels. These SOX10-bound enhancer and super-enhancer regions are associated with genes that affect pigmentation as well as the development and differentiation of neural crest and melanocytes, consistent with SOX10 functioning at these loci in re-activation of pigmentation and developmental pathways under MAPK inhibition in 501mel cells. These results for SOX10 in melanocytes correlate with previous data showing other SOX family members can bind to super-enhancers and regulate lineage-specific genes in chondrocytes and hair follicle stem cells [37, 38]. Strikingly, the super-enhancer-associated genes bound by SOX10 included *MITF, TFAP2A, ETS1, ZEB2,* and *SNAI2,* each lineage-specific transcription factors known to play important roles in melanocyte development and pigmentation [41, 66, 67, 96–100]. Several of these factors exhibit co-binding and/or regulation of one another during neural crest and melanocyte development; for example, ETS1 and SOX10 interact to promote proper melanocyte development [40, 99], TFAP2A and MITF interact to regulate pigmentation [54, 100], and SOX10 activates MITF, while MITF has been suggested to have the capacity to both activate and repress SOX10 expression [74–77, 101, 102]. SOX10 binding at super-enhancers near these genes suggests SOX10 is integral to the complex regulatory cross talk exhibited by these lineage-determining transcription factors. Interestingly, the partial knockdown of SOX10 did not prevent the AZD6244-induced appearance of pigmentation in 501mel cells; this may reflect the ability of the remaining SOX10 protein to still undergo genomic recruitment or possible compensation by other cellular mechanisms.

SOX10 recognizes either monomeric or palindromic dimer motifs (as do the other SOXE family members SOX8 and SOX9) and causes substantial bending of the DNA helix via binding to the minor groove, a characteristic shared by all SOX proteins [40, 103–106]. Our consensus motif profile data from SOX10 ChIP showed enrichment of both monomer and dimer binding profiles, suggesting the potential for monomeric and dimeric SOX10 binding at H3K27ac-marked enhancers. Interestingly, SOX10 monomer and dimer binding causes different angles of DNA bending (70°–85° or 101°, respectively, [103]). Bending of enhancer DNA has been suggested to regulate enhancer structure and transcriptional regulation, and furthermore, specific bending angles may facilitate differential recruitment of transcriptional cofactors, as has been shown for SOX2 [95, 107]. Future functional studies will be needed to determine if monomeric and dimeric binding of SOX10 at these H3K27ac-marked enhancers confers distinct functional properties. SOX10 is known to act at enhancers to recruit the SWI/SNF chromatin remodeling complex protein BRG1 in melanocytes [18] and also to recruit the mediator complex (which is critical for recruitment of RNA Polymerase II) via binding to MED12 in oligodendrocytes and Schwann cells [108]. While further cofactors that interact with SOX10 at DNA have been suggested in glial cells [109, 110], the extent of SOX10 cofactor recruitment and interaction remains under-explored in melanocytes.

SOX proteins generally confer transcriptional regulation by co-binding with additional transcription factors [111], and candidate SOX10-interacting transcription factors in melanocytes are suggested by the significantly enriched consensus motifs present at the SOX10 ChIP peaks identified in this study. Under both DMSO and AZD6244 treatment, consensus motifs were found for HBP1 and LEF1, critical transcription factors for WNT/B-catenin signaling [112–114], as well as E-box motifs for the MITF/TFE family of basic helix-loop-helix (bHLH) transcription factors. Co-localization of consensus sequences and chromatin binding for MITF and SOX10 has been observed previously in melanocytes [40, 41]. In agreement with these findings, a comparison of our SOX10 ChIP binding with previously identified MITF binding sites in 501mel cells [115] found that 42.7% of our SOX10 peaks colocalize with MITF binding regions. This suggests the opportunity for cooperative binding and synergistic action of MITF and SOX10, similar to what has been observed at the DCT promoter [78, 79]. Additionally, novel SOX10-interacting candidates are suggested by the presence of consensus motifs for FOXG1, FOXJ2, and FOXJ3, factors whose roles in melanocyte development or function have not been analyzed to date.

GWAS studies have identified over 800 SNPs associated with human pigmentation variation and melanocyte-related phenotypes [84–86]. Most GWAS SNPs reside in non-coding regions [19, 82, 83] and are hypothesized to reside in cis-regulatory regions or in close linkage disequilibrium (LD) to causative SNPs located within cis-regulatory regions. Therefore, GWAS results implicate the region of LD surrounding each SNP in affecting the associated phenotype, and subsequent analysis is needed to directly ascertain causative alleles and/or haplotypes. The large number of SNPs associated with human pigmentation reflects variation in GWAS studies performed in diverse populations, the identification of two or more distinct SNPs within the same LD region by different studies, multiple enhancers conferring regulation at the same genomic locus, and the polygenic nature of pigmentation itself. Integration of our epigenetic roadmap with GWAS variation found that 90 of these pigmentation/melanoma GWAS SNPs are located within SOX10 binding sites and/or enhancers that undergo dynamic changes under MEK inhibition, highlighting SNPs contained within these regions as intriguing candidates for future analyses of enhancer regulatory regions affecting pigmentation. The variants (and their respective LD regions) identified in this study provide an epigenetic link between the MAPK pathway, variation in normal human pigmentation, and melanoma susceptibility, thus potentially expanding the applicability of these data from 501mel cells to a broad range of melanocyte phenotypes, with potential implications for disease progression in MAPK-related disorders and drug responses that alter MAPK signaling.

## Conclusions

This study reveals the extensive genomic responses that result from MEK inhibition in 501mel melanoma cells, including widespread remodeling of the active chromatin landscape, increased recruitment of SOX10 binding to the genome, and re-activation of melanocyte pigmentation and developmental transcriptional programs. Beyond specific mechanistic insight into the MEK inhibitor response in BRAF(V600E) mutant melanoma cells, this study provides a comprehensive genomic resource for future studies to identify potential novel loci for pigmentation and drug resistance in melanocytes and melanoma.

## Methods

### Cell culture and Western blot

The human melanoma cell line 501mel (kindly provided by Dr. Yardena Samuels) was cultured in RPMI 1640 medium supplemented with 10% fetal bovine serum (FBS) and DMEM, respectively. The human melanoma cell line A375 (ATCC, Manassas, VA) and the mouse melanoma cell line B16F10 (ATCC) were cultured in DMEM medium supplemented with 10% FBS. All cell lines were maintained at 37 °C under 5% CO_2_/95% humidified air. For shSOX10 knockdown, stable transfections of either a non-targeting shRNA or two independent shSOX10 constructs were performed in triplicate; then, the cells were subjected to puromycin selection for 2 weeks. All shRNA constructs (non-targeting shRNA: catalog # RHS6848; shSOX10-2: catalog# RHS3979-201750192 and shSOX10-5: catalog # RHS3979-201750195) were purchased from Dharmacon (Lafayette, CO). All cell lines were treated with DMSO (vehicle control) or 200 nM AZD6244 (MEK inhibitor) for 48 h (Western blot analyses of phosphorylated ERK) or 72 h (all studies). Western blots were performed according to standard protocols.

### ChIP-Seq and peak calling

SOX10 and H3K27ac ChIP-Seq assays were performed essentially as described previously [40, 116, 117]. All ChIP-Seq data were derived from two independent experimental replicates of DMSO-treated and AZD6244-treated 501mel cells. Total genomic DNA (input) derived from formaldehyde cross-linked samples was used as control for normalization during peak calling. Raw sequence reads that passed quality control were aligned to the human reference genome GRCh37/hg19 (available from the UCSC genome browser, http://genome.ucsc.edu/) using Burrows Wheeler Alignment (BWA; [118]. Peak calling on all ChIP-Seq data was performed using MACS v2.1 [119] by applying the default settings and significance thresholds (format = AUTO (fragment length detection from libraries), model fold = [10, 100], *q* value cutoff = 5.00e−02 or FDR < 0.05), and using input genomic DNA as background control.

### Differential binding analysis of ChIP-Seq data

After identifying ChIP-Seq peaks with MACS2, significantly differentially bound regions across samples were detected by quantification of H3K27ac ChIP-Seq tag density using the Bioconductor software package DiffBind [57]; http://bioconductor.org/packages/release/bioc/html/DiffBind.html), which was implemented in R as follows. MACS detected peaks that overlapped in at least two libraries, i.e., only replicated peaks, were retained by setting minOverlaps = 2 in the dba.count function of DiffBind, and all binding sites identified as overlapping were merged. In order to identify peaks with significant differences in read densities (differential binding affinity), we first obtained “consensus peaks” as follows: (1) overlapping peaks between DMSO and AZD6244 were merged and the largest 5′ to 3′ footprint was retained; (2) regions with no overlaps were also retained and included in the consensus peaks to enable analysis of peaks that are only present in one sample. Next, the number of reads within each experimental condition overlapping the consensus peaks was counted, and the overlapping reads from the corresponding genomic background (input) were subtracted. Then, contrasts were made between the treatment conditions and significantly differentially enriched regions were determined by implementing DESeq2 within DiffBind, which performs a normalization procedure based on trimmed mean of *M* values (TMM) using the overall depth of sequencing in each library (ChIP read counts minus input read counts and full library size) and calculates enrichment *p* values. High-confidence differentially enriched regions were obtained by correcting *p* values for multiple testing using the Benjamini–Hochberg procedure, and only sites with an FDR < 0.1 were retained for downstream analysis.

### Identification of super-enhancers

Super-enhancers were identified using the rank ordering of super-enhancers (ROSE) algorithm (http://younglab.wi.mit.edu/super_enhancer_code.html, [31]). Briefly, peaks were called on H3K27ace ChIP-Seq bam files using MACS2 as described above using the human reference genome GRCh37/hg19 (see peak-calling procedure). Peaks located within 2500 base pairs (bp) of TSSs were excluded, as a 5-kb TSS exclusion zone was applied. The remaining peaks were designated as putative enhancers. Enhancers located within 12,500 base pairs of each other were stitched together, scored, and ranked based on the H3K27ac ChIP-Seq signal (total normalized read count in the ChIP-Seq sample after input subtraction). Then, enhancer signal (H3K27ac density) was plotted against enhancer rank. Enhancer regions above the inflection point of the curve were designated as super-enhancers, and those below the inflection point were designated as typical enhancers. Super-enhancers were assigned to the nearest RefSeq gene or genes using the 3 default parameters of the ROSE algorithm: included were overlapping genes (transcript directly overlapping the super-enhancer), proximal genes (TSS within 50 kb of the center of the super-enhancer), and nearest gene.

### Motif discovery

H3K27ac ChIP-Seq regions were scanned for both de novo and known motifs using the findMotifsGenome.pl functionality in HOMER (Hypergeometric Optimization of Motif EnRichment, v4.6, http://homer.ucsd.edu/homer/index.html), with the default settings and whole genome as background [62]. For de novo motif analysis of super-enhancer regions, a 1000-bp sequence surrounding the center of each H3K27ac site was searched for enriched motifs using HOMER. SOX10 ChIP-Seq regions, defined by the peak summit and including ± 100 bp, were evaluated for both de novo and known motifs using MEME-ChIP [120].

### RNA extraction and RNA-Seq data analysis

501mel cells were harvested with TRIzol reagent (Invitrogen/Thermo Fisher Scientific, Inc.) followed by RNA extraction using the Direct-zol™ RNA Miniprep Kit (Zymo Research). RNA quality was assessed by Bioanalyzer (Agilent Technologies, Inc.) and samples that passed the quality control metric (RIN > 6.5) were used for RNA-Seq. Paired-end poly-A enriched RNA-Seq libraries were generated by the NIH Intramural Sequencing Center (NISC) using an Illumina TruSeq small RNA kit from ~ 200 ng total RNA. The RNA samples were sequenced to generate at least 54 million 101-base read pairs for each individual library. Following quality filtering, RNA-Seq reads were mapped to the hg19 genome with STAR [121] and differential expression analysis of genes was performed using DESeq2 ([122], https://bioconductor.org/packages/release/bioc/html/DESeq2.html). Further RNA-Seq read quality control and calculation of number of mapped reads were performed using RSeQC [123] and RSEM [124], respectively. The normalized expression and differentially expressed genes between DMSO treatment and AZD6244 treatment conditions were identified using DESeq2 with an FDR of 0.1. Only nonzero tag counts in either condition were considered for further analysis.

### Pigment gene identification

The 250 pigmentation genes identified in this study have been described in a previously published list of 650 genes that show visible integument pigment phenotypes in human, mouse, or zebrafish [51]. The 250 pigmentation genes were identified by determining the overlap of the 638 human orthologs contained within this published list with our gene lists. Of note, the visible pigmentation phenotypes associated with the 650 genes include both increased and decreased pigmentation, developmental abnormalities affecting melanocyte specification and survival, and pigmentation in adults, such as graying.

## Additional files

**Additional file 1: Figure S1.** MEK inhibition induces hyperpigmentation in mouse B16F10 and human A375 melanoma cell lines. Cells were treated with 200 nM AZD6244 for 72 h. Cell suspensions are shown above cell pellets, with equal numbers of cells present in samples with and without AZD6244 treatment. Note that the B16F10 cells are pigmented initially, and then exhibited a further darkening of pigmentation under AZD6244 treatment. **Figure S2.** Enriched biological processes identified by GREAT analyses on the 16,476 regions that were losing H3K27ac and the 1041 regions that were gaining H3K27ac under AZD6244 treatment. **Figure S3.** Effect of MEK inhibition on super-enhancers in 501mel cells. A) Distribution of H3K27ac ChIP-Seq signal at enhancers (input-subtracted reads per million per enhancer regions) in AZD6244-treated 501mel cells. Enhancers are ranked by decreasing H3K27ac ChIP-Seq signal; the gray dashed line demarcates the boundary between super-enhancers (red) and typical enhancers (gray). Of the six super-enhancer-associated transcription factors with motifs enriched in 501mel super-enhancer regions (Fig. 3b), four (*MITF*, *SOX10*, *ETS1* and *ETV5*) persisted as super-enhancer-associated under AZD6244 treatment. B) Box-and-scatter plots of super-enhancer signal density (rpm/bp) in control (SE_DMSO) and AZD6244 treated (SE_AZD) 501mel cells. Significance of the difference between distributions determined using a two-tailed t test. ^***^p value ≤ 0.001. **Figure S4.** SOX10 shows increased genome-wide binding under AZD6244 treatment, including recruitment to H3K27ac-marked regions. The numbers of SOX10 binding sites from SOX10 ChIP analysis throughout the genome and in relation to H3K27ac-marked regions in 501mel cells are shown, both under control (DMSO-treated) and AZD6244-treated conditions. **Figure S5.** Significantly enriched transcription factor motifs identified under SOX10 ChIP-Seq peaks. Enriched motifs are shown for (A) control (DMSO-treated) conditions and (B) AZD6244-treated conditions. **Figure S6.** Transcription factors that regulate melanocyte lineage and pigmentation processes undergo significant expression changes under AZD6244 and are associated with SOX10-bound super-enhancers. UCSC genome browser tracks are shown for the transcription factors *MITF, ETS1, ZEB2, SNAI2,* and *TFAP2A*. Colored tracks indicate control and AZD6244-treated regions with H3K27ac binding (pink and gray), with differentially acetylated H3K27ac levels (orange and brown), super-enhancers (light and dark green), and SOX10-ChIP genomic binding (teal and blue). Note the novel recruitment of SOX10 to the super-enhancer regions associated with these genes resulting from AZD6244 treatment (teal sites that do not overlap blue sites). **Figure S7.** Partial reduction of SOX10 protein using shRNA knockdown in 501mel cells does not prevent the ability of AZD6244 to induce pigmentation. We performed stable knockdowns in triplicate experiments using two different shSOX10 constructs (shSOX10 #2 and shSOX10 #5), and these cells were then subjected to either DMSO or AZD6244 treatment for 72 h. Western blotting for SOX10, phospho-ERK and GAPDH (loading control) showed that each shSOX10 induced a partial knockdown of SOX10 protein, and AZD6244 treatment reduced phosphorylated ERK levels. Visual inspection of the cells indicated that while all cells under DMSO treatment remained unpigmented (indicated by “−” sign), pigmentation was induced under AZD6244 treatment for all cells (indicated by “+” sign), including in the context of shSOX10 treatment. NT = non-targeting.

**Additional file 2: Table S1.** Genes exhibiting significantly different expression changes in 501mel cells in response to AZD6244 treatment. **Table S2.** The 250 pigmentation genes with differential expression in 501mel cells under AZD6244 treatment. Genes associated with H3K27ac loss or gain regions by GREAT analysis, or with SOX10 binding sites under AZD6244 are also indicated. **Table S3.** Top 5 significantly enriched transcription factor motifs identified by de novo motif enrichment analysis of genomic regions that were losing or gaining H3K27ac in 501mel cells in response to AZD6244 treatment. **Table S4.** The 799 super-enhancers identified in 501mel cells treated with DMSO only, and the 583 super-enhancers identified in 501mel cells treated with AZD6244. **Table S5.** Top enriched biological processes from GO analyses of 1080 genes associated with super-enhancers in control (DMSO-treated) 501mel cells. **Table S6.** Super-enhancer-associated pigmentation genes with significant expression changes following AZD6244 treatment. **Table S7.** GWAS loci associated with pigmentation and/or melanoma phenotypes that overlap with H3K27ac regions and/or with SOX10 binding sites.

## Data Availability

ChIP-Seq (H3K27ac and SOX10) and RNA-Seq datasets of 501mel samples supporting the conclusions of this article have been deposited to the NCBI Sequence Read Archive (SRA) database (https://www.ncbi.nlm.nih.gov/sra/) under the Bioproject Accession Number PRJNA515302.
