# Supplementary material for: MEK inhibition remodels the active chromatin landscape and induces SOX10 genomic recruitment in BRAF(V600E) mutant melanoma cells
Source: Epigenetics Chromatin. 2019 Aug 9;12:50. doi: 10.1186/s13072-019-0297-2 (PMC6688322; doi:10.1186/s13072-019-0297-2)
Supplement: Supplementary file 1 — Additional file 1: Figure S1. MEK inhibition induces hyperpigmentation in mouse B16F10 and human A375 melanoma cell lines. Cells were treated with 200 nM AZD6244 for 72 h. Cell suspensions are shown above cell pellets, with equal numbers of cells present in samples with and without AZD6244 treatment. Note that the B16F10 cells are pigmented initially, and then exhibited a further darkening of pigmentation under AZD6244 treatment. Figure S2. Enriched biological processes identified by GREAT analyses on the 16,476 regions that were losing H3K27ac and the 1041 regions that were gaining H3K27ac under AZD6244 treatment. Figure S3. Effect of MEK inhibition on super-enhancers in 501mel cells. A) Distribution of H3K27ac ChIP-Seq signal at enhancers (input-subtracted reads per million per enhancer regions) in AZD6244-treated 501mel cells. Enhancers are ranked by decreasing H3K27ac ChIP-Seq signal; the gray dashed line demarcates the boundary between super-enhancers (red) and typical enhancers (gray). Of the six super-enhancer-associated transcription factors with motifs enriched in 501mel super-enhancer regions (Fig. 3b), four (MITF, SOX10, ETS1 and ETV5) persisted as super-enhancer-associated under AZD6244 treatment. B) Box-and-scatter plots of super-enhancer signal density (rpm/bp) in control (SE_DMSO) and AZD6244 treated (SE_AZD) 501mel cells. Significance of the difference between distributions determined using a two-tailed t test. ***p value ≤ 0.001. Figure S4. SOX10 shows increased genome-wide binding under AZD6244 treatment, including recruitment to H3K27ac-marked regions. The numbers of SOX10 binding sites from SOX10 ChIP analysis throughout the genome and in relation to H3K27ac-marked regions in 501mel cells are shown, both under control (DMSO-treated) and AZD6244-treated conditions. Figure S5. Significantly enriched transcription factor motifs identified under SOX10 ChIP-Seq peaks. Enriched motifs are shown for (A) control (DMSO-treated) conditions and (B) AZD6244- [file 13072_2019_297_MOESM1_ESM.pptx]

## Slide 1
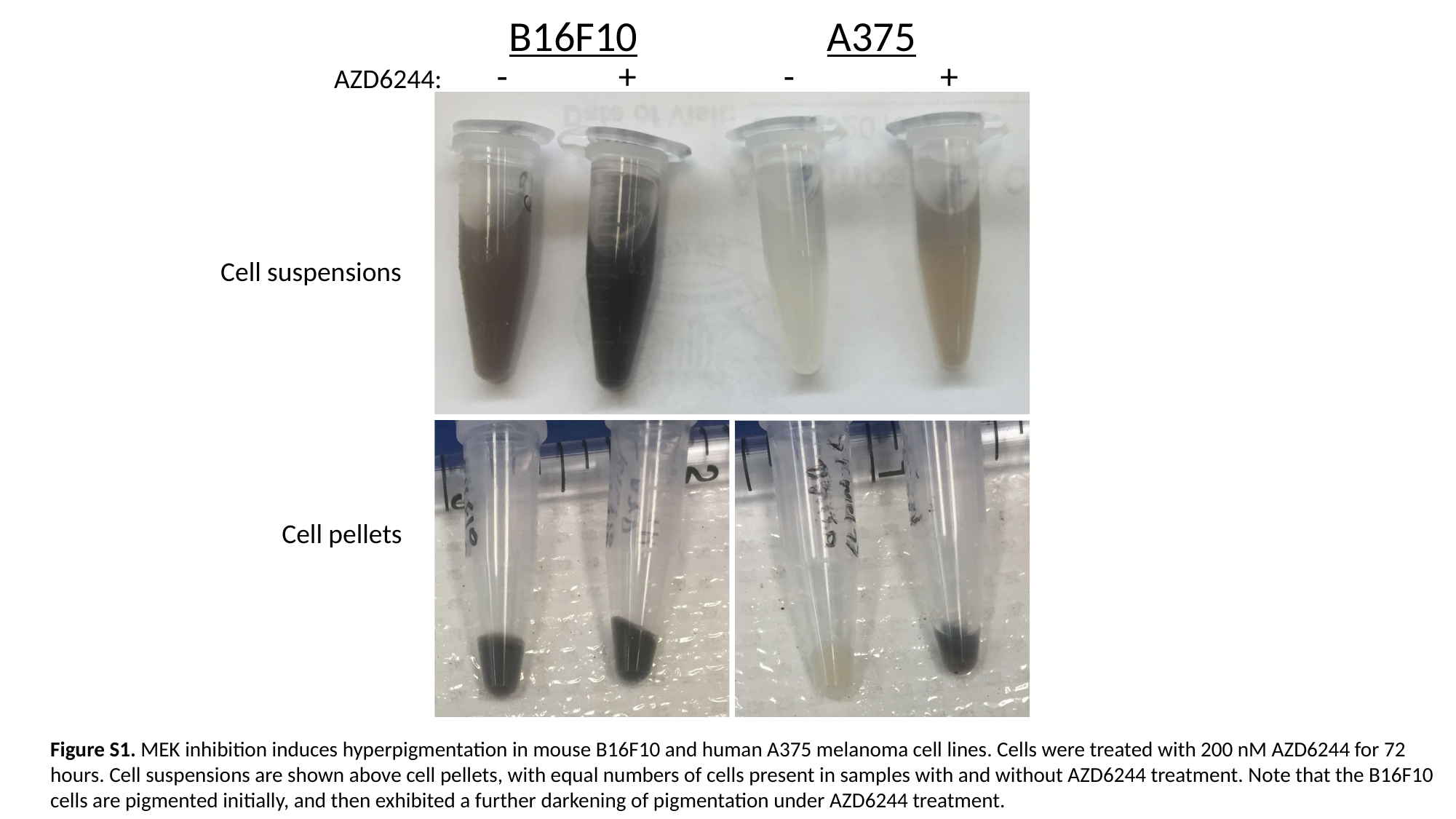

B16F10
A375
-
+
-
+
AZD6244:
Cell suspensions
Cell pellets
Figure S1. MEK inhibition induces hyperpigmentation in mouse B16F10 and human A375 melanoma cell lines. Cells were treated with 200 nM AZD6244 for 72 hours. Cell suspensions are shown above cell pellets, with equal numbers of cells present in samples with and without AZD6244 treatment. Note that the B16F10 cells are pigmented initially, and then exhibited a further darkening of pigmentation under AZD6244 treatment.

## Slide 2
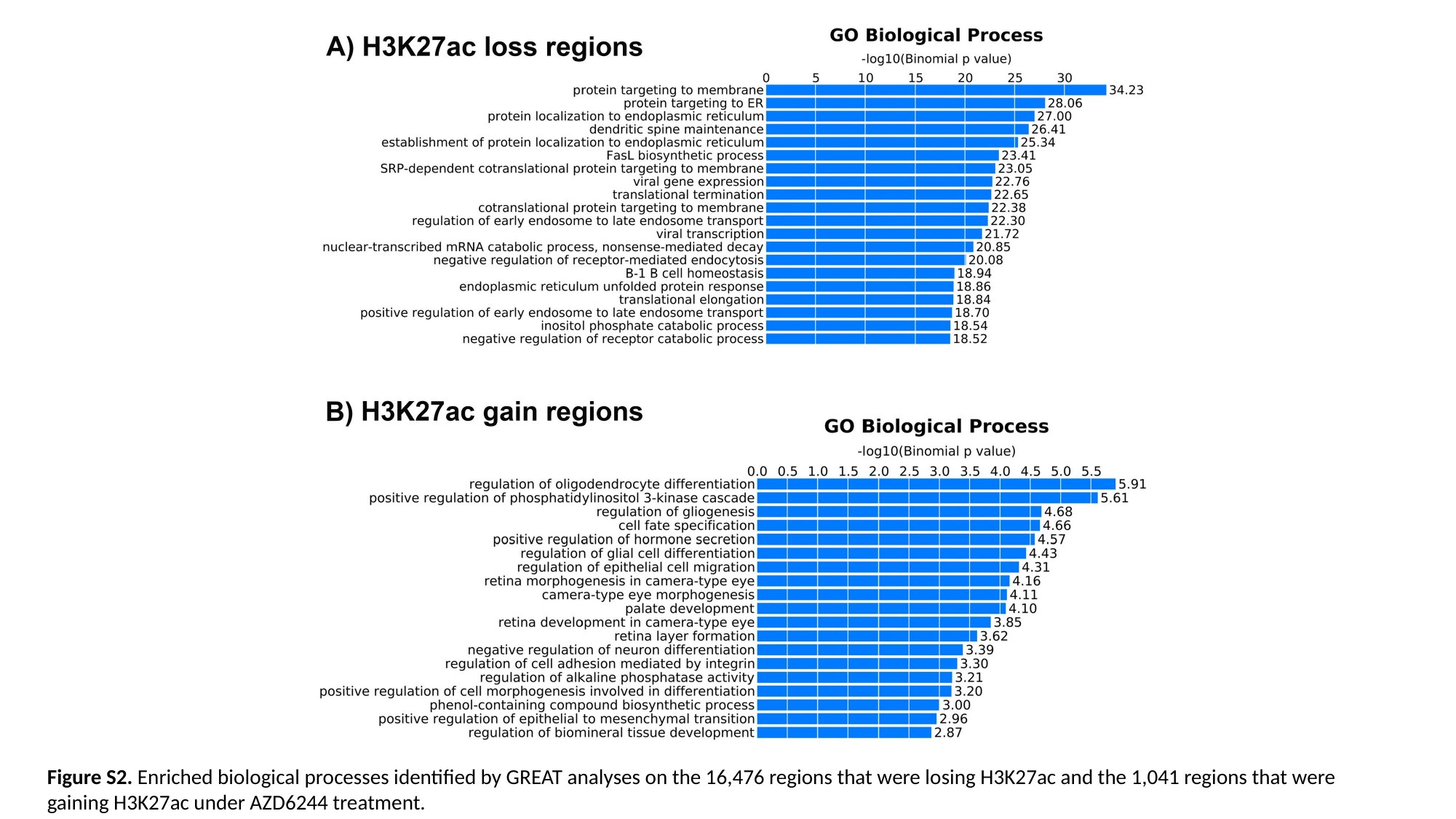

Figure S2. Enriched biological processes identified by GREAT analyses on the 16,476 regions that were losing H3K27ac and the 1,041 regions that were gaining H3K27ac under AZD6244 treatment.

## Slide 3
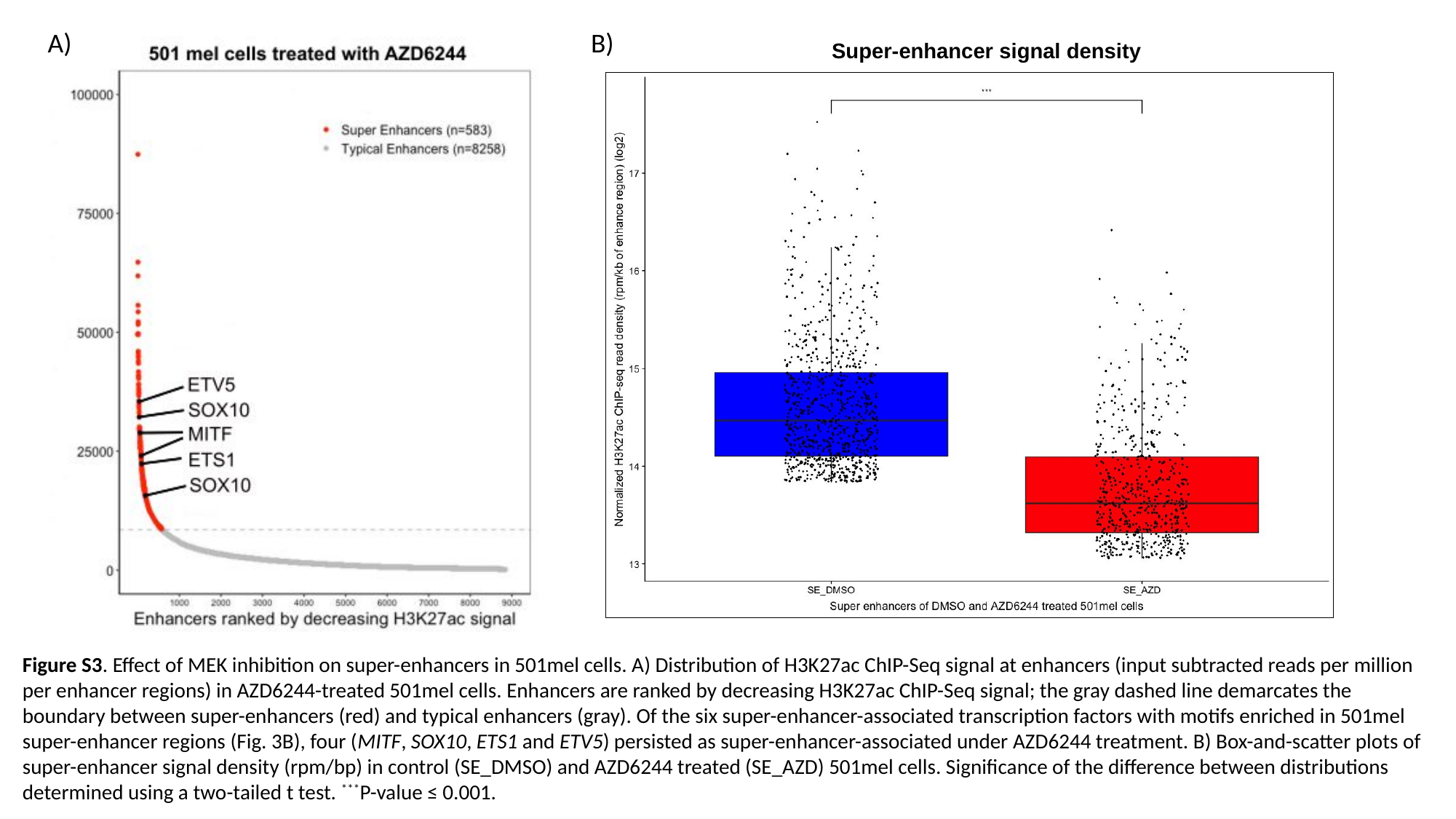

A)
B)
Super-enhancer signal density
Figure S3. Effect of MEK inhibition on super-enhancers in 501mel cells. A) Distribution of H3K27ac ChIP-Seq signal at enhancers (input subtracted reads per million per enhancer regions) in AZD6244-treated 501mel cells. Enhancers are ranked by decreasing H3K27ac ChIP-Seq signal; the gray dashed line demarcates the boundary between super-enhancers (red) and typical enhancers (gray). Of the six super-enhancer-associated transcription factors with motifs enriched in 501mel super-enhancer regions (Fig. 3B), four (MITF, SOX10, ETS1 and ETV5) persisted as super-enhancer-associated under AZD6244 treatment. B) Box-and-scatter plots of super-enhancer signal density (rpm/bp) in control (SE_DMSO) and AZD6244 treated (SE_AZD) 501mel cells. Significance of the difference between distributions determined using a two-tailed t test. ***P-value ≤ 0.001.

## Slide 4
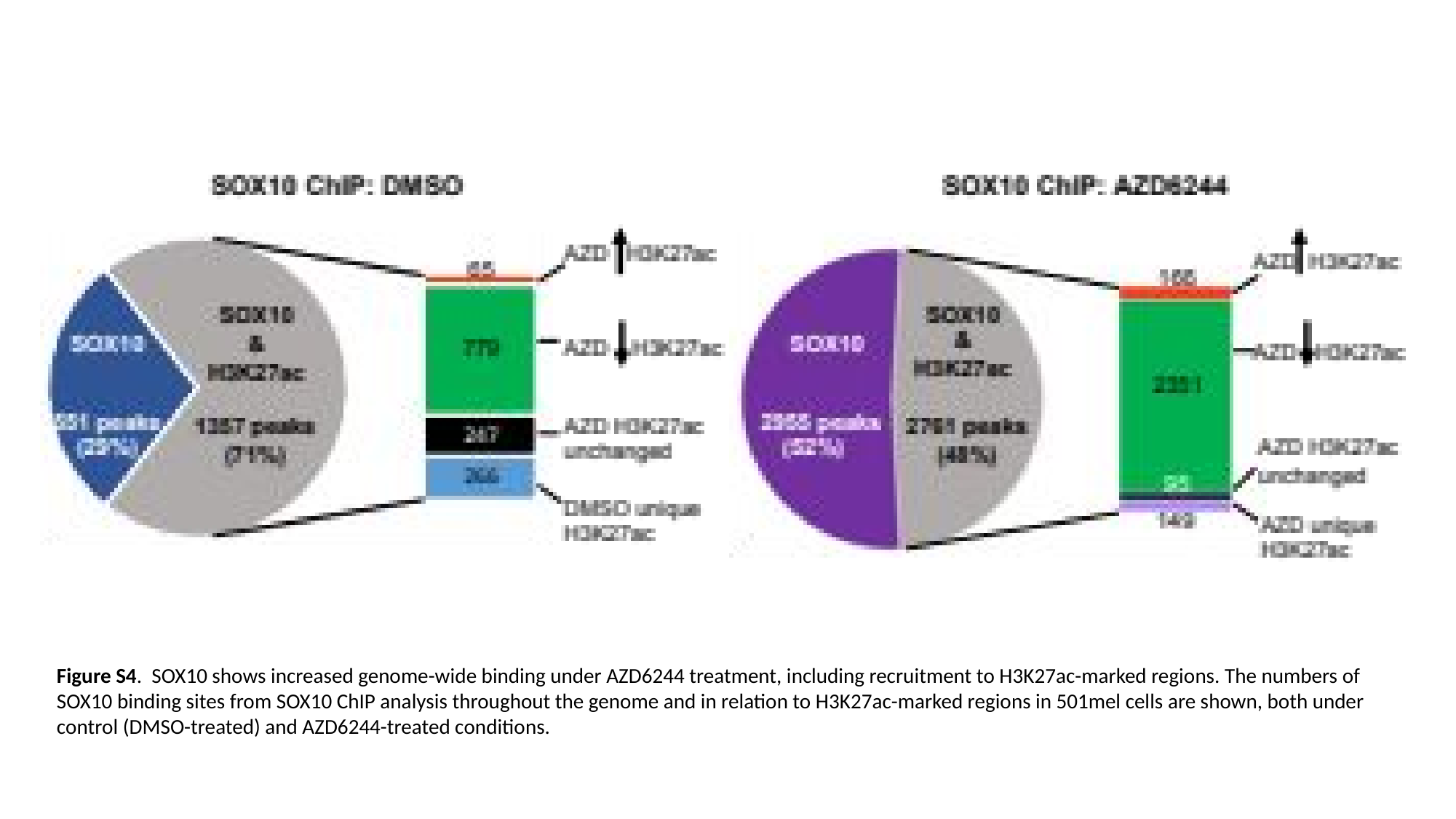

Figure S4. SOX10 shows increased genome-wide binding under AZD6244 treatment, including recruitment to H3K27ac-marked regions. The numbers of SOX10 binding sites from SOX10 ChIP analysis throughout the genome and in relation to H3K27ac-marked regions in 501mel cells are shown, both under control (DMSO-treated) and AZD6244-treated conditions.

## Slide 5
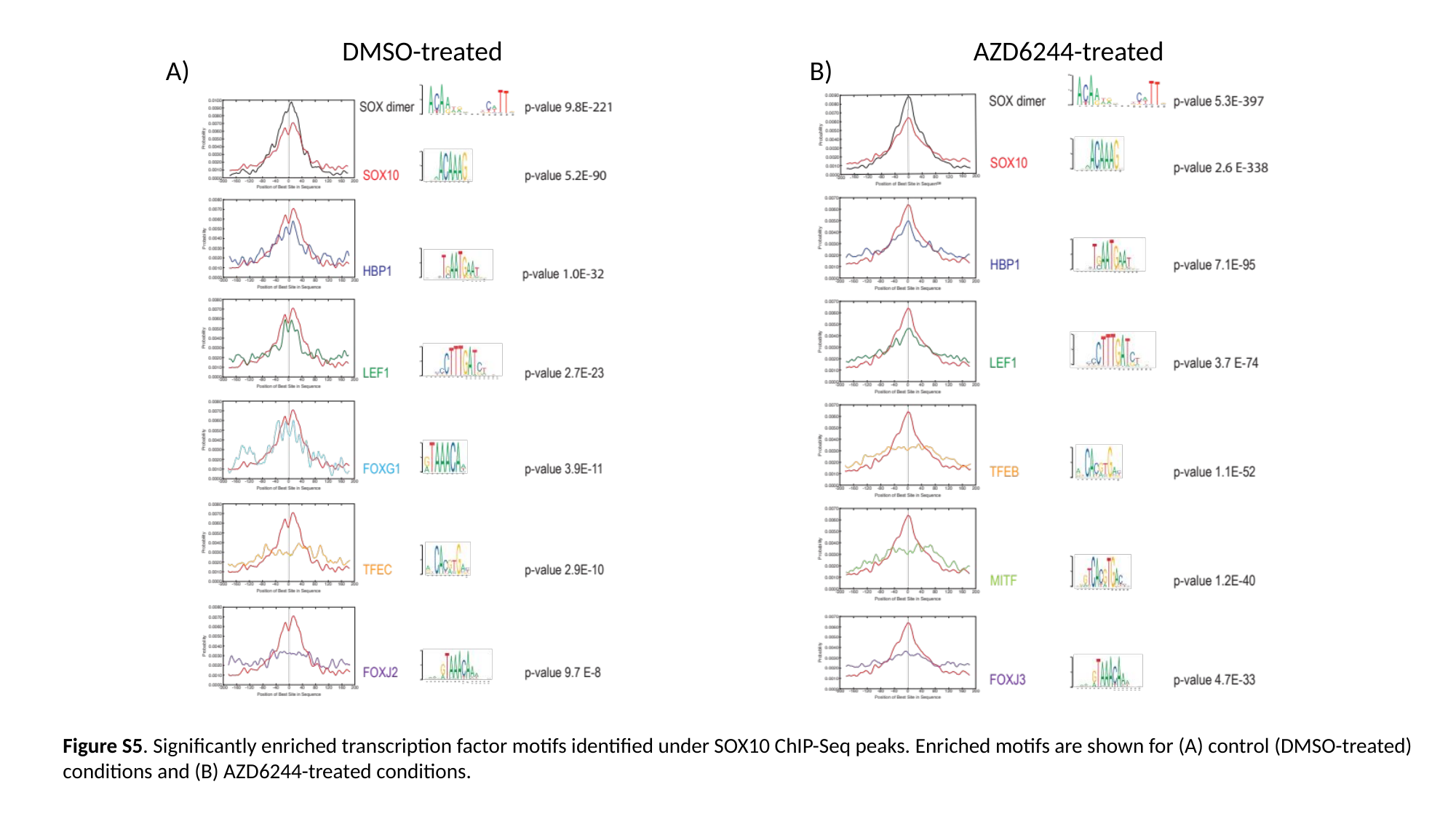

DMSO-treated
AZD6244-treated
A)
B)
Figure S5. Significantly enriched transcription factor motifs identified under SOX10 ChIP-Seq peaks. Enriched motifs are shown for (A) control (DMSO-treated) conditions and (B) AZD6244-treated conditions.

## Slide 6
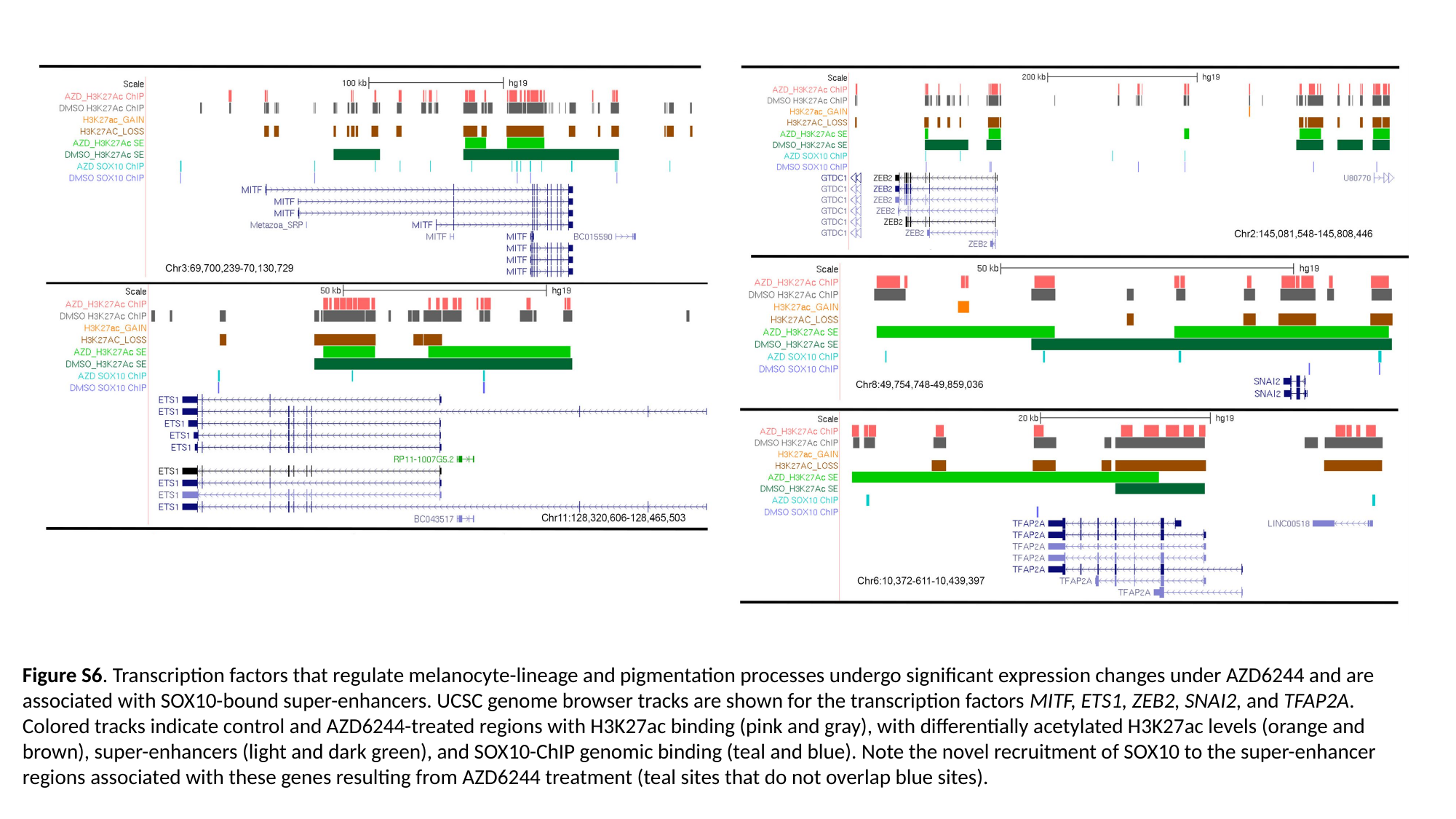

Figure S6. Transcription factors that regulate melanocyte-lineage and pigmentation processes undergo significant expression changes under AZD6244 and are associated with SOX10-bound super-enhancers. UCSC genome browser tracks are shown for the transcription factors MITF, ETS1, ZEB2, SNAI2, and TFAP2A. Colored tracks indicate control and AZD6244-treated regions with H3K27ac binding (pink and gray), with differentially acetylated H3K27ac levels (orange and brown), super-enhancers (light and dark green), and SOX10-ChIP genomic binding (teal and blue). Note the novel recruitment of SOX10 to the super-enhancer regions associated with these genes resulting from AZD6244 treatment (teal sites that do not overlap blue sites).

## Slide 7
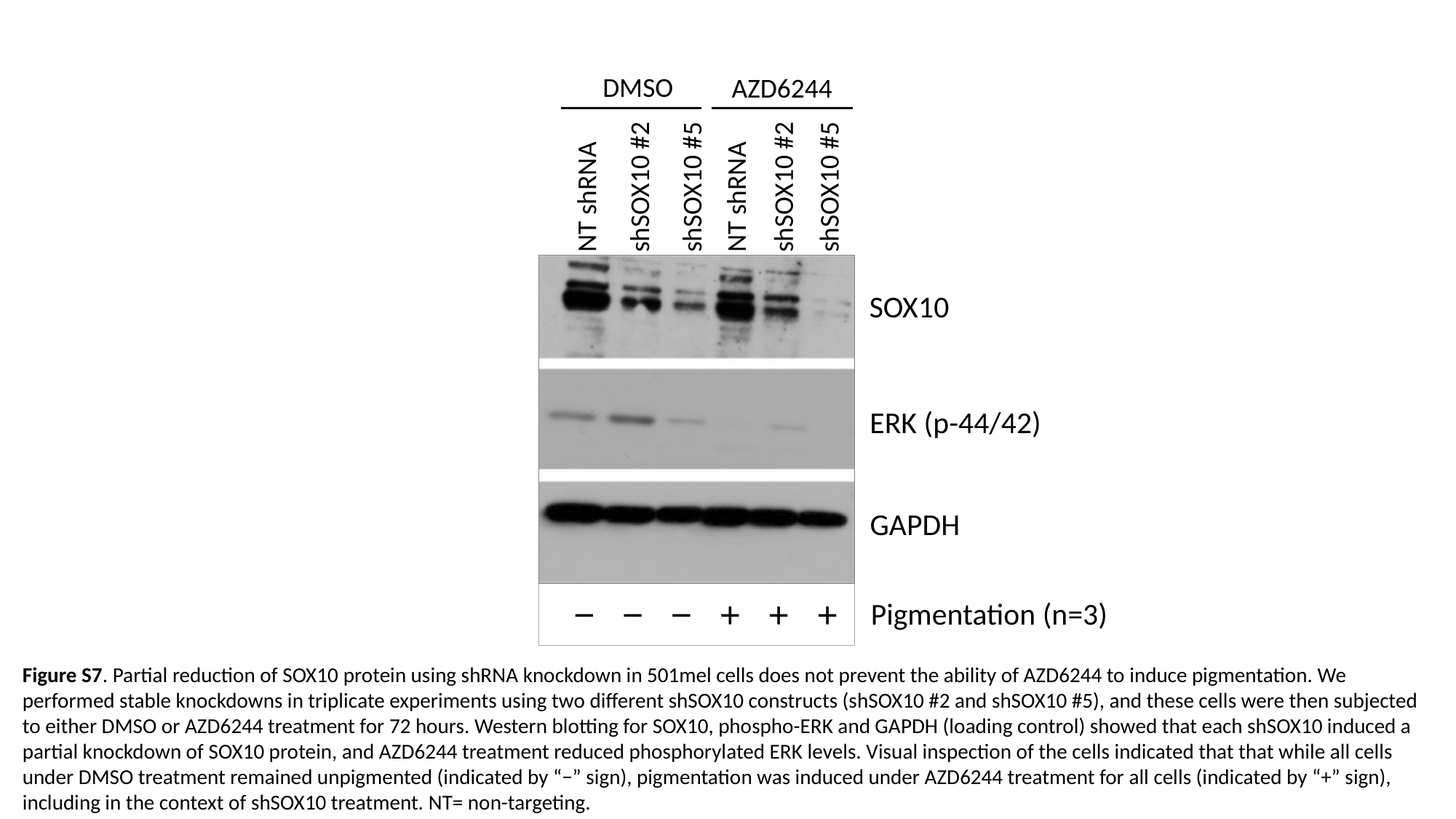

DMSO
AZD6244
shSOX10 #2
shSOX10 #5
shSOX10 #2
shSOX10 #5
NT shRNA
NT shRNA
SOX10
ERK (p-44/42)
GAPDH
−
−
−
+
+
+
Pigmentation (n=3)
Figure S7. Partial reduction of SOX10 protein using shRNA knockdown in 501mel cells does not prevent the ability of AZD6244 to induce pigmentation. We performed stable knockdowns in triplicate experiments using two different shSOX10 constructs (shSOX10 #2 and shSOX10 #5), and these cells were then subjected to either DMSO or AZD6244 treatment for 72 hours. Western blotting for SOX10, phospho-ERK and GAPDH (loading control) showed that each shSOX10 induced a partial knockdown of SOX10 protein, and AZD6244 treatment reduced phosphorylated ERK levels. Visual inspection of the cells indicated that that while all cells under DMSO treatment remained unpigmented (indicated by “−” sign), pigmentation was induced under AZD6244 treatment for all cells (indicated by “+” sign), including in the context of shSOX10 treatment. NT= non-targeting.
